# Supplementary material for: Chymotrypsin is a molecular target of insect resistance of three corn varieties against the Asian corn borer, Ostrinia furnacalis
Source: PLoS One. 2022 Apr 8;17(4):e0266751. doi: 10.1371/journal.pone.0266751 (PMC8992986; doi:10.1371/journal.pone.0266751)
Supplement: S1 Table — (DOCX) [file pone.0266751.s001.docx]

**S1 Table. Artificial diet (1 L) treatments containing three different corn varieties: Ilmichal (IM), Kwangpyeongok (KP), and P3394**

| Components | Control diet | IM diet | KP diet | P3394 diet |
| --- | --- | --- | --- | --- |
| Wheat germ (g) | 40.3 | 8.1 | 8.1 | 8.1 |
| Corn leaf powder (g) | 0.0 | 32.2 | 32.2 | 32.2 |
| Casein (g) | 34.1 | 34.1 | 34.1 | 34.1 |
| Glucose (g) | 31.0 | 31.0 | 31.0 | 31.0 |
| Vitamin mixture (g) | 7.1 | 7.1 | 7.1 | 7.1 |
| Salt mixture (g) | 11.2 | 11.2 | 11.2 | 11.2 |
| β-Sitosterol (g) | 2.5 | 2.5 | 2.5 | 2.5 |
| Ascorbic acid (g) | 9.3 | 9.3 | 9.3 | 9.3 |
| Methyl-ρ-hydroxybenzoate (g) | 1.6 | 1.6 | 1.6 | 1.6 |
| Fumidil B (500ppm) (g) | 0.5 | 0.5 | 0.5 | 0.5 |
| Aureomycin (g) | 2.1 | 2.1 | 2.1 | 2.1 |
| Propionic- phosphoric acid (g) | 6.7 | 6.7 | 6.7 | 6.7 |
| Sorbic acid (g) | 0.6 | 0.6 | 0.6 | 0.6 |
| Agar (g) | 20.2 | 20.2 | 20.2 | 20.2 |
| Distilled water (mL) | 1,000 | 1,000 | 1,000 | 1,000 |
