# Supplementary material for: Chymotrypsin is a molecular target of insect resistance of three corn varieties against the Asian corn borer, Ostrinia furnacalis
Source: PLoS One. 2022 Apr 8;17(4):e0266751. doi: 10.1371/journal.pone.0266751 (PMC8992986; doi:10.1371/journal.pone.0266751)
Supplement: S2 Table — (DOCX) [file pone.0266751.s002.docx]

**S2 Table. List of primers used in this study**

| Primer | Uses | Sequence (5’ – 3’) | AT (℃) | Size (bp) |
| --- | --- | --- | --- | --- |
| OfCHY 1 | RT-PCR | 5′-GGA ACT ACT CCA ATG ATG TT-3′  5′-GAA TAT GTA TTC TTC ACC GT-3′ | 54 | 251 |
| OfCHY 2 | RT-PCR | 5′-ATA TAA ACA ACG ACA TCG CA-3′  5′-AAT GAA ATT AGG AAA CGC CT-3′ | 50 | 239 |
| OfCHY 3 | RT-PCR | 5′-CTA GAC CCA TTA ATC TAC CAA-3′  5′-AAC ACC AAT CAG AAT ACG G-3′ | 54 | 293 |
| OfCHY 4 | RT-PCR | 5′-TAT TCT CAA ATA GGG TAC AGC-3′  5′-CGT TTT CGA CAA GAG GA-3′ | 50 | 270 |
| OfCHY 5 | RT-PCR | 5′-AAA GAG GCG CAT AAG AAC-3′  5′-CGT CAT CGT ATA GAG TTT TC-3′ | 50 | 226 |
| OfCHY 6 | RT-PCR | 5′-GAC CAA GTG ATT TTA TCT GG-3′  5′-TTT TTA ATT AGT GGT CCT CCT-3′ | 54 | 212 |
| RL32 | RT-PCR | 5′-GTC CTC ATC ACC TCC TCA AAC-3′  5′-CAG AGT CAC CGT TGC AAG TA-3′ | 54 | 270 |
| dsCHY3_T7 | RNAi | 5′-TAA TAC GAC TCA CTA TAG GCA ACG ATT TCG CTG GAT C-3′  5′-TAA TAC GAC TCA CTA TAG GGA GAG CTC CGA ACG AAA CAA CAC-3′ | 56 | 765 |

‘AT’ stands for annealing temperature.
