# Supplementary material for: Chymotrypsin is a molecular target of insect resistance of three corn varieties against the Asian corn borer, Ostrinia furnacalis
Source: PLoS One. 2022 Apr 8;17(4):e0266751. doi: 10.1371/journal.pone.0266751 (PMC8992986; doi:10.1371/journal.pone.0266751)
Supplement: S3 Table — (DOCX) [file pone.0266751.s003.docx]

**S3 Table. Putative chymotrypsin (CHY) genes of *O. furnacalis***

| Gene | DNA (bp) | Protein (AA) | Annotation (species) | Accession No. |
| --- | --- | --- | --- | --- |
| *OfCHY1* | 1,216 | 298 | CHY BI-like (*O. furnacalis*) | XP_028173785.1 |
| *OfCHY2* | 926 | 283 | CHY1-like (*O. furnacalis*) | XP_028178136.1 |
| *OfCHY3* | 1,327 | 283 | CHY1-like (*O. furnacalis*) | XP_028168554.1 |
| *OfCHY4* | 1,115 | 254 | CHY2-like (*O. furnacalis*) | XP_028170065.1 |
| *OfCHY5* | 866 | 277 | CHY1-like (*O. furnacalis*) | XP_028164682.1 |
| *OfCHY6* | 1,169 | 245 | CHY2-like (*O. furnacalis*) | XP_028164700.1 |
| *OfCHY7* | 1,170 | 246 | CHY2-like (*O. furnacalis*) | XP_028164699.1 |
| *OfCHY8* | 2,507 | 365 | CHY-like (*O. furnacalis*) | XP_028169758.1 |
| *OfCHY9* | 856 | 282 | CHY2-like (*O. furnacalis*) | XP_028173191.1 |
