# Supplementary material for: Chymotrypsin is a molecular target of insect resistance of three corn varieties against the Asian corn borer, Ostrinia furnacalis
Source: PLoS One. 2022 Apr 8;17(4):e0266751. doi: 10.1371/journal.pone.0266751 (PMC8992986; doi:10.1371/journal.pone.0266751)
Supplement: S4 Table — (DOCX) [file pone.0266751.s004.docx]

**S4 Table. GenBank accession numbers and abbreviations used for phylogenetic analysis**

| *Species* | *Abbreviate* | *Gene name* | *Accession No.* |
| --- | --- | --- | --- |
| *Aedes aegypti* | Aa | chymotrypsin -like serine protease (JA15) | AY957559 |
|  | Aa L | Larval chymotrypsin-like | AF487334 |
| *Anopheles gambiae* | Ag | chymotrypsin | Z18887 |
| *Culex pipiens pallens* | CpP | Putative chymotrypsin-like | AF468495 |
| *Glossina morsitans* | Gm | Morsitans chymotrypsin-like | EU589385 |
| *Lutzomyia longipalpis* | Ll chym 1A | Putative chymotrypsin | EU124576 |
|  | Ll chym 1B | Putative chymotrypsin | EU124575 |
|  | Ll chym 2 | Putative chymotrypsin | EU124583 |
|  | Ll chym 3 | Putative chymotrypsin | EU124591 |
|  | Ll chym 4 | Putative chymotrypsin | EU124573 |
|  | Ll chym 5 | Putative chymotrypsin | EU124574 |
| *Phlebotomus papatasi* | Pp chym 1 | Chymotrypsin (chym1) | AY128106 |
|  | Pp chym 2 | Chymotrypsin (chym2) | AY128107 |
| *Stomoxys calcitrans* | Sc | chymotrypsin | AY190632 |
| *Agrotis ipsilon* | Ai | AiC5 chymotrypsinogen | AAF71516 |
| *Bombyx mori* | Bm P | Chymotrypsin-like proteinase | JQ081296 |
|  | Bm | Chymotrypsin-like | NM_001046965 |
| *Danaus plexippus* | Dp | Chymotrypsin | EHJ67193 |
| *Diatraea saccharalis* | Ds | Chymotrypsin 2b | AFW03966 |
| *Helicoverpa armigera* | Ha | Clone HaFLS01825 chymotrypsin | EU325550 |
| *Helicoverpa punctigera* | Hp | chymotrypsinogen | AAV33655 |
| *Helicoverpa zea* | Hz | HzC4 chymotrypsinogen | AAF71518 |
| *Heliothis virescens* | Hv | chymotrypsin | AFM28261 |
| *Mythimna separata* | Ms | chymotrypsin | AKR06192.1 |
| *Ostrinia nubilalis* | On | Midgut chymotrypsin | AFM77775.1 |
| *Papilio machaon* | Pm | Chymotrypsin | KPJ01456 |
| *Papilio xuthus* | Px | chymotrypsin | KPJ18047 |
| *Spodoptera frugiperda* | Sf | Chymotrypsin precursor | AY251276 |
| *Spodoptera litura* | Sl CTLP2 | Chymotrypsin-like protein 2 (CTLP2) | GQ891130 |
|  | Sl | Chymotrypsin-like protein | GQ354838 |
| *Anoplophora glabripennis* | Ag chym | chymotrypsin | XP_023312143.1 |
| *Phaedon cochleariae* | Pc | chymotrypsin | CAA76928.1 |
| *Tribolium castaneum* | Tc1 | Chymotrypsin BI | XP_968239.2 |
|  | Tc | Chymotrypsin-like proteinase 5A precursor | CBC01177.1 |
| *Ostrinia nubilalis* | On CHY | Chymotrypsin-like serine protease 16 | AFM77775 |
| *Bombyx mori* | Bm CHY | Chymotrypsin-like proteinase | AFD99127 |
| *Manduca sexta* | Ms CHY | Chymotrypsin-like protein 3 | CAM84318 |
| *Papilio xuthus* | Px CHY | Chymotrypsin-2 | KPI90986 |
